# Inhibition of CYP1A1 Alleviates Colchicine-Induced Hepatotoxicity

**DOI:** 10.3390/toxins16010035

**Published:** 2024-01-09

**Authors:** Ruoyue Huang, Jingyi Duan, Wen Huang, Yan Cheng, Beiwei Zhu, Fei Li

**Affiliations:** 1 Department of Gastroenterology & Hepatology, Laboratory of Metabolomics and Drug-Induced Liver Injury, State Key Laboratory of Biotherapy, and Frontiers Science Center for Disease-Related Molecular Network, West China Hospital, Sichuan University, Chengdu 610041, China; 2 Laboratory of Ethnopharmacology, Tissue-Orientated Property of Chinese Medicine Key Laboratory of Sichuan Province, West China School of Medicine, West China Hospital, Sichuan University, Chengdu 610041, China; 3 Academician Workstation, Jiangxi University of Chinese Medicine, Nanchang 330004, China; 4 School of Food Science and Technology, Dalian Polytechnic University, Dalian 116034, China; zhubeiwei@163.com; 5 National Engineering Research Center of Seafood, Dalian 116034, China; 6 State Key Laboratory of Respiratory Health and Multimorbidity, West China Hospital, Sichuan University, Chengdu 610041, China

**Keywords:** colchicine, CYP1A1, oxidative stress, pyroptosis, metabolism

## Abstract

Colchicine, a natural compound extracted from Colchicum autumnale, is a phytotoxin, but interestingly, it also has multiple pharmacological activities. Clinically, colchicine is widely used for the treatment of gouty arthritis, familial Mediterranean fever, cardiovascular dysfunction and new coronary pneumonia. However, overdose intake of colchicine could cause lethal liver damage, which is a limitation of its application. Therefore, exploring the potential mechanism of colchicine-induced hepatotoxicity is meaningful. Interestingly, it was found that CYP1A1 played an important role in the hepatotoxicity of colchicine, while it might also participate in its metabolism. Inhibition of CYP1A1 could alleviate oxidative stress and pyroptosis in the liver upon colchicine treatment. By regulating CYP1A1 through the CASPASE-1-GSDMD pathway, colchicine-induced liver injury was effectively relieved in a mouse model. In summary, we concluded that CYP1A1 may be a potential target, and the inhibition of CYP1A1 alleviates colchicine-induced liver injury through pyroptosis regulated by the CASPASE-1-GSDMD pathway.

## 1. Introduction

Colchicine, a natural compound extracted from *Colchicum autumnale*, is a phytotoxin, but interestingly, it also has multiple pharmacological activities and therefore has been used for the treatment of various diseases [1]. As it possesses good anti-inflammatory properties, it was used to treat gout for a long time, and familial Mediterranean fever for years. Recently, its protective effect on cardiovascular disease and COVID-19 was also proved by clinical studies [2,3,4]. However, although colchicine has a wide application prospect, it causes serious side effects in vital organs such as the liver, intestine and kidney [5]. In addition to overdose intake, there are other factors that cause poisoning. For example, *C. autumnale* is sometimes eaten by mistake because it is hard to distinguish from traditional edible plants, such as *Allium ursinum* or bear’s garlic in Middle Europe [6]. The narrow therapeutic indicators and the lethal high dose of colchicine greatly limit its wide application in clinics [7]. Therefore, it is important to explore the mechanism of its toxicity and search for strategies to improve the safety of its clinical use.

As a member of the major cytochrome P450 (CYP) enzymes in the liver, CYP1A1 has been comprehensively studied for its ability to metabolize exogenous and endogenous compounds because of its carcinogenic derivatives [8]. Previous studies have shown that CYP1A1 is associated with liver injuries such as non-alcoholic fatty liver disease [9]. CYP1A1 is a monooxygenase whose activation can lead to the overproduction of reactive oxygen species (ROS) that lead to the result of oxidative stress [10], which usually occurs in a damaged liver. Colchicine is a well-known substrate for P-glycoprotein (P-gp) and CYP3A4, and P-gp is widely distributed in tissues, participates in the transport of colchicine and is closely related to toxicity [11,12]. Studies on the metabolism of colchicine showed that the CYP3A4 enzyme is the major metabolic enzyme and plays a role in reducing liver toxicity [13,14]. However, whether CYP1A1 plays a role in the hepatotoxicity induced by colchicine is unknown.

It was found that the regulation of CYP1A1 could effectively alleviate colchicine-induced liver injury through the CASPASE-1-GSDMD pathway and therefore provided a potential therapeutic target for colchicine-induced liver injury. Caution should be taken when using colchicine combined with drugs targeting CYP1A1 in the future.

## 2. Results

### 2.1. Liver Injury Induced by Colchicine in Mice

**Figure 1:**
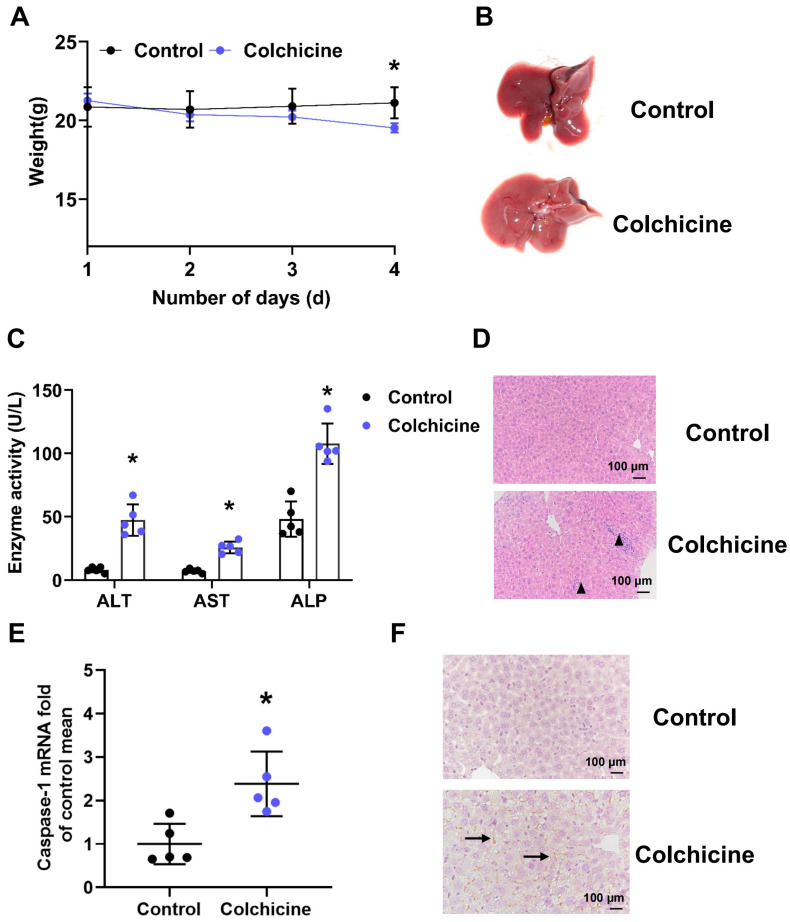
Colchicine induced liver injury in a dose-dependent manner. (**A**) The body weight of mice decreased significantly in the colchicine group. (**B**) Gross anatomy of the liver after colchicine treatment. (**C**) After colchicine management, aspartate aminotransferase (AST), alanine aminotransferase (ALT) and alkaline phosphatase (ALP) enzyme levels were significantly increased. (**D**) Liver inflammatory injury after colchicine management as observed using H&E staining is shown by black triangles. (**E**) Gene expression level of Caspase-1 in the liver after colchicine treatment. (**F**) Immunohistochemical staining of CASPASE-1 in the liver. All data are expressed as mean ± SD (*n* = 5). * *p* < 0.05 was considered statistically significant, compared with the control group.

Colchicine could cause liver toxicity in mice, mainly manifested in increased enzyme activity, inflammatory injury and hepatocyte pyroptosis in a dose-dependent manner (Figure 1 and Figure S1). Phenotypically, the weight of mice treated with colchicine decreased significantly (Figure 1A). The gross anatomy of the liver showed an enlarged liver and shrunken gallbladder in the colchicine group (Figure 1B). An inflamed liver was observed using H&E staining in the colchicine group (Figure 1C). Upon colchicine administration, ALT, AST and ALP enzyme levels in plasma were significantly increased (Figure 1D). Colchicine activated CASPASE-1, which is closely related to pyroptosis (Figure 1E,F). The results showed that colchicine caused liver injury in mice.

### 2.2. CYP1A1 Metabolizes Colchicine

**Figure 2:**
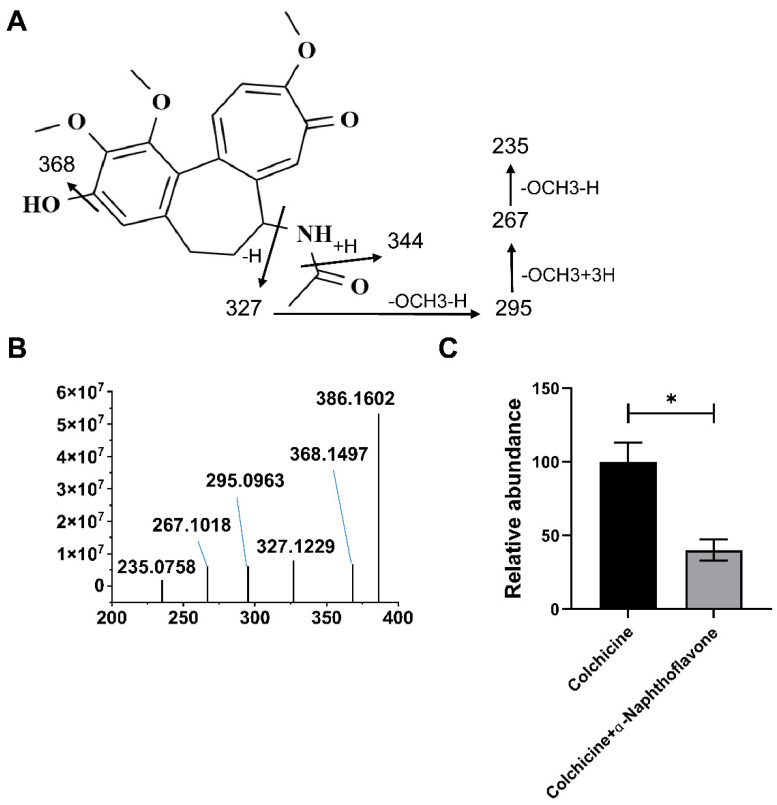
CYP1A1 is involved in the metabolism of colchicine. (**A**) Structural formula for colchicine demethylated metabolite. (**B**) MS/MS of colchicine demethylated metabolite. (**C**) Demethylating metabolism was inhibited after administration of α-naphthoflavone. All data are expressed as mean ± SD (*n* = 3). * *p* < 0.05 was considered statistically significant, compared with the control group.

The results of colchicine metabolism by the CYP recombinase enzyme revealed that the modification and metabolism of colchicine by CYP recombinase enzymes mainly occurred at the demethylene group (M2−M6), as shown in Table 1. The metabolic map of colchicine is shown in Figure S2. The CYP recombinases (CYP1A2, CYP2C19, CYP1B1, CYP2D6, CYP2A6, CYP2C8, CYP2B6, CYP2C9, CYP3A4, CYP1A1, CYP3A5, CYP2E1 and CYP4A11) were involved in the processes of the metabolism of colchicine, such as demethylation, dehydrogenation, and so on, and CYP1A1 was involved in the metabolism of colchicine. The structure of the demethylated metabolite was confirmed by MS/MS analysis (Figure 2A,B). The demethylation metabolism of colchicine by CYP1A1 was significantly inhibited by the administration of α-naphthoflavone, a CYP1A1 inhibitor (Figure 2C). The results showed that CYP1A1 plays a role in the metabolism of colchicine.

### 2.3. CYP1A1 Was Significantly Elevated by Colchicine

**Figure 3:**
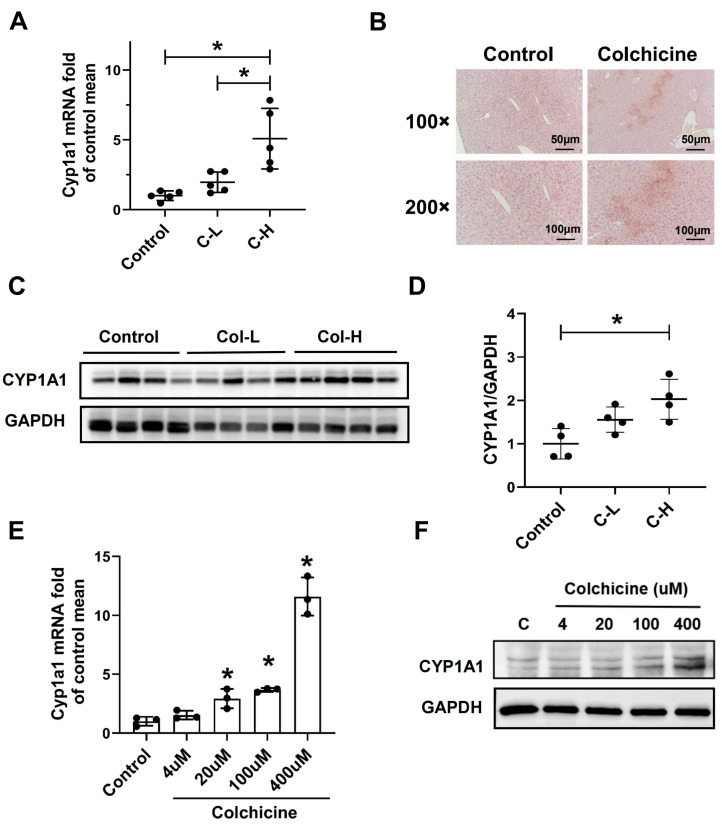
CYP1A1 was significantly elevated after colchicine treatment. (**A**) Gene expression level of CYP1A1 in the liver. (**B**) Immunohistochemical staining of CYP1A1 in the liver. (**C**) Colchicine increased the level of CYP1A1 protein expression in the liver. (**D**) In the liver, total protein was normalized, and relative intensities of immunoblot of CYP1A1 are shown as a bar graph. In primary hepatocytes, gene expression level of CYP1A1 (**E**) and protein expression level of CYP1A1 (**F**). All data are expressed as mean ± SD (*n* = 5). * *p* < 0.05 was considered statistically significant, compared with the control group.

The demethylation metabolism of colchicine has been reported to reduce toxicity, and this study found that CYP1A1 was involved in the demethylation metabolism of colchicine. However, CYP1A1 has not yet been reported as associated with colchicine-induced hepatotoxicity; therefore, we investigated the gene and protein expression of CYP1A1. Colchicine increased both the mRNA and protein levels of the CYP1A1 enzyme in the liver in a dose-dependent manner (Figure 3A–D). Also, in primary hepatocytes, colchicine dose-dependently increased the mRNA and protein levels of CYP1A1 (Figure 3E,F). These results showed that CYP1A1 was significantly elevated in the colchicine model.

### 2.4. Regulation of CYP1A1 Could Regulate Colchicine-Induced Liver Injury

**Figure 4:**
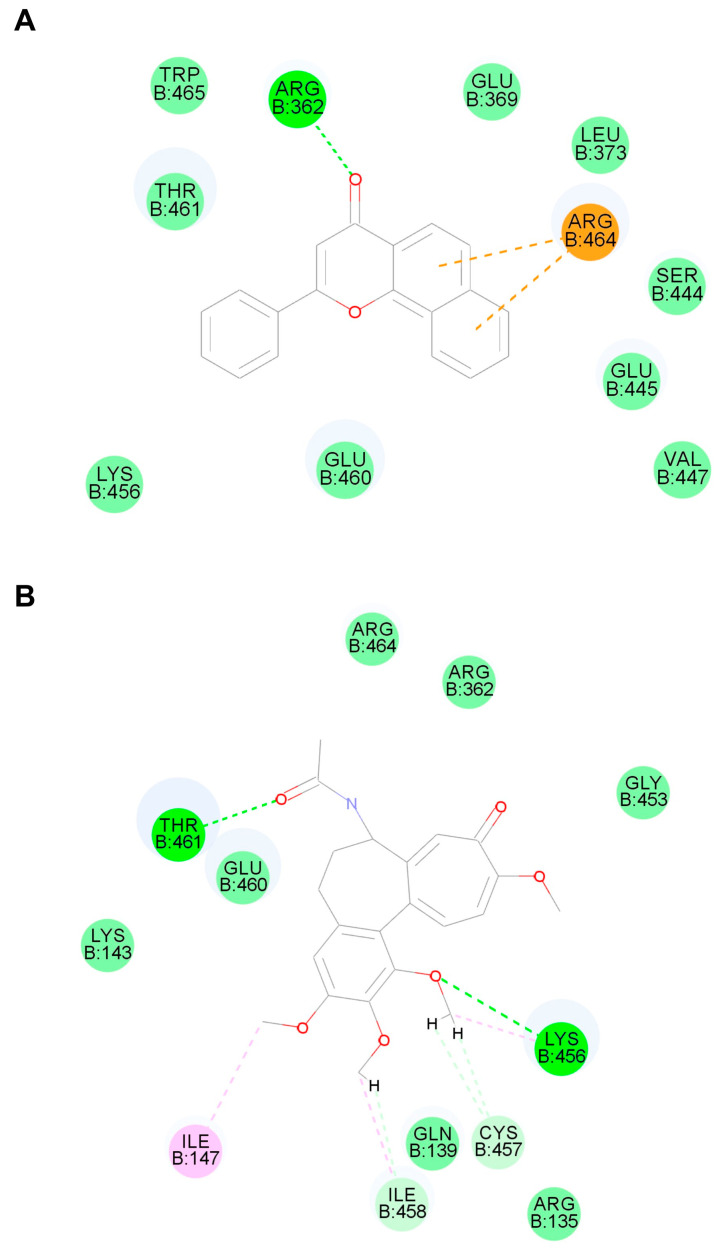
Molecular docking results of CYP1A1 and colchicine (**A**) or α-naphthoflavone (**B**). Green dashed line indicates conventional hydrogen bonds, light green dashed line indicates van der Waals, orange dashed line indicates pi–cation and pink dashed line indicates alkyl.

As a monooxygenase, CYP1A1 is not only involved in the metabolism of drugs, but also closely related to liver damage. Overproduction of reactive oxygen species (ROS) is related to its activation and eventually causes oxidative stress [10]. Previous literature has shown that modulating CYP1A1 has significant effects on a variety of organ injuries [15]. The present study found that CYP1A1 was significantly elevated in the colchicine model, yet whether it played a role in colchicine-induced liver injury is still unknown. Using CYP1A1 inhibitor α-naphthoflavone as a reference compound, molecular docking showed that colchicine could bind to the CYP1A1 enzyme (Figure 4). So, it is necessary to study the role of CYP1A1 in colchicine-induced liver injury.

**Figure 5:**
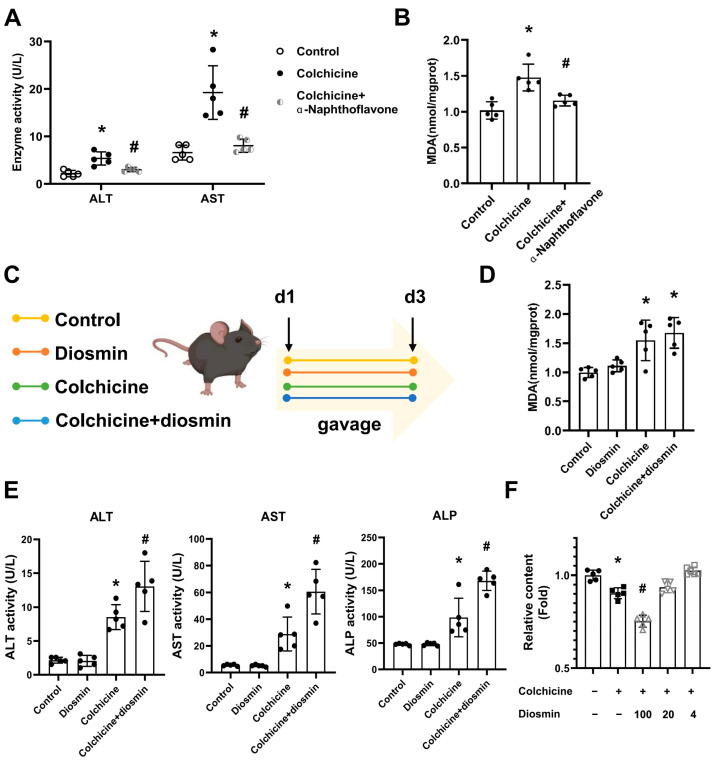
Colchicine-induced liver injury could be relieved by regulating the level of CYP1A1. (**A**) α-naphthoflavone could improve colchicine-induced liver injury, indicated by the activity of aspartate aminotransferase (AST) and alanine aminotransferase (ALT). (**B**) Effects of α-naphthoflavone on the malondialdehyde (MDA) level in the liver after colchicine management. (**C**) Procedure to investigate the effect of CYP1A1 on colchicine-induced liver injury. (**D**) The level of MDA in the liver after mice were co-treated with colchicine and diosmin management. (**E**) AST, ALT and alkaline phosphatase (ALP) enzyme activities were significantly increased after mice were co-treated with colchicine and diosmin. (**F**) The results of MTT in primary hepatocytes after cells were co-treated with colchicine and diosmin. All data are expressed as mean ± SD (*n* = 5); * *p* < 0.05 and # *p* < 0.05 were considered statistically significant when compared with the control group and colchicine group, respectively.

α-naphthoflavone significantly alleviated liver injury that was induced by colchicine, as shown in the improvement of enzyme activity and oxidative stress (Figure 5A,B). Reversely, the CYP1A1 inducer diosmin effectively aggravated colchicine-induced hepatotoxicity (Figure 5C–E). Diosmin exacerbated primary hepatocyte death caused by colchicine (Figure 5F). The results indicated that regulation of CYP1A1 might significantly change the liver damage induced by colchicine.

### 2.5. Inhibition of CASPASE-1-Mediated Pyroptosis Pathway by Inhibiting CYP1A1

**Figure 6:**
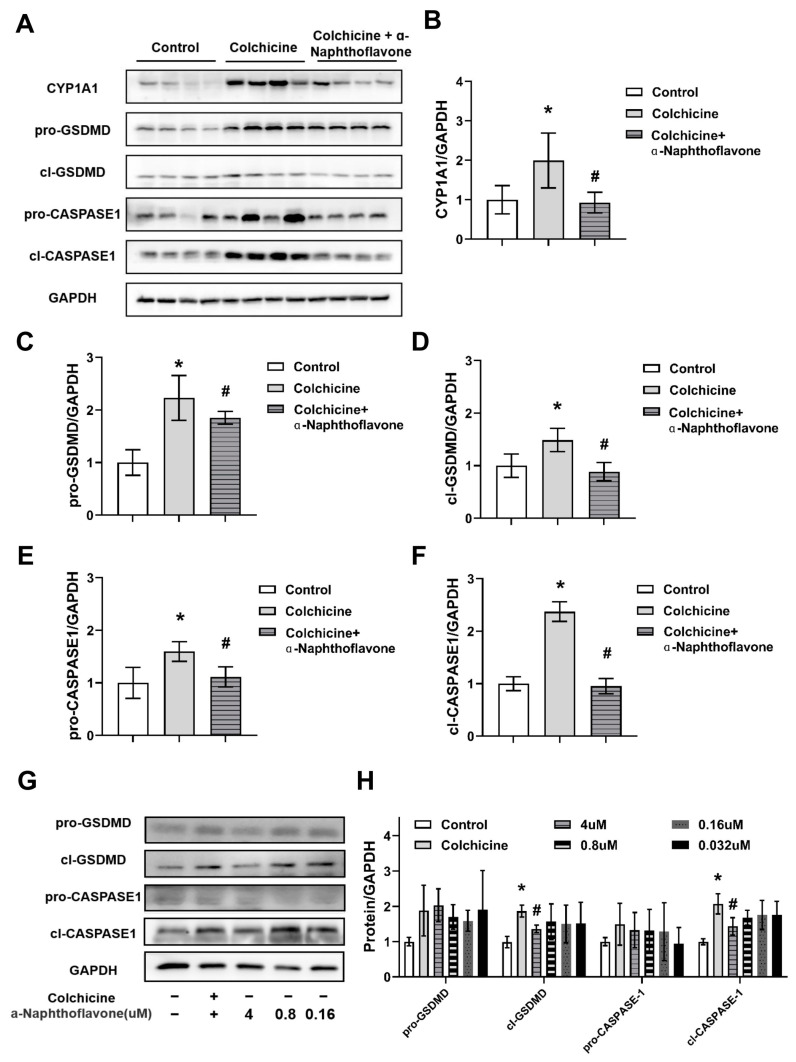
Inhibition of CASPASE-1-mediated pyroptosis pathway by inhibiting CYP1A1. Colchicine activated the expression of the CASPASE-1-GSDMD pathway, while α-naphthoflavone down-regulated these proteins in the liver (**A**). Relative intensities of immunoblots of CYP1A1 (**B**), pro-GSDMD (**C**), cl-GSDMD (**D**), pro-CASPASE1 (**E**) and cl-CASPASE1 (**F**). In the liver, total protein was normalized, and the results are shown as a bar graph. (**G**) WB results of CASPASE-1-GSDMD pathway in primary hepatocytes after management with colchicine and different concentrations of α-naphthoflavone. (**H**) In primary hepatocytes, the total protein was normalized, and the relative intensities of the immunoblot of the CASPASE-1-GSDMD pathway are shown as a bar graph for the colchicine or colchicine + α-naphthoflavone group. All data are expressed as mean ± SD (*n* = 5); * *p* < 0.05 and # *p* < 0.05 were considered statistically significant when compared with the control group and colchicine group, respectively.

Previous studies suggested many factors can cause liver damage, including pyroptosis and oxidative stress [16,17]. Moreover, there are several signal pathways related to pyroptosis, and the CASPASE-1-GSDMD pathway is the classical pathway [18]. In the present study, we found that colchicine activated CASPASE-1 in the liver and colchicine-induced liver injury was alleviated by regulating CYP1A1, but whether CYP1A1 plays a role through regulating CASPASE-1-GSDMD-mediated pyroptosis remains unknown. To further determine the potential downstream consequence of CYP1A1 modulation in colchicine-induced liver injury, mice were co-treated with colchicine and the CYP1A1 inhibitor α-naphthoflavone. The colchicine-activated CASPASE-1-GSDMD-mediated pyroptosis was restored upon α-naphthoflavone treatment (Figure 6). Colchicine activated the CASPASE-1-GSDMD pathway while α-naphthoflavone significantly down-regulated these proteins in the liver, as shown by the elevated protein levels of cl-CASPASE and cl-GSDMD (Figure 6A–F). The CASPASE-1-GSDMD pathway was significantly up-regulated in the colchicine group, and α-naphthoflavone restored the abnormal protein levels in a dose-gradient manner in primary hepatocytes (Figure 6G,H). Taken together, these results indicate that the inhibition of CYP1A1 can alleviate liver injury induced by colchicine possibly through the CASPASE-1-GSDMD pathway.

### 2.6. Aggravation of CASPASE-1-Mediated Pyroptosis Pathway by Activating CYP1A1

**Figure 7:**
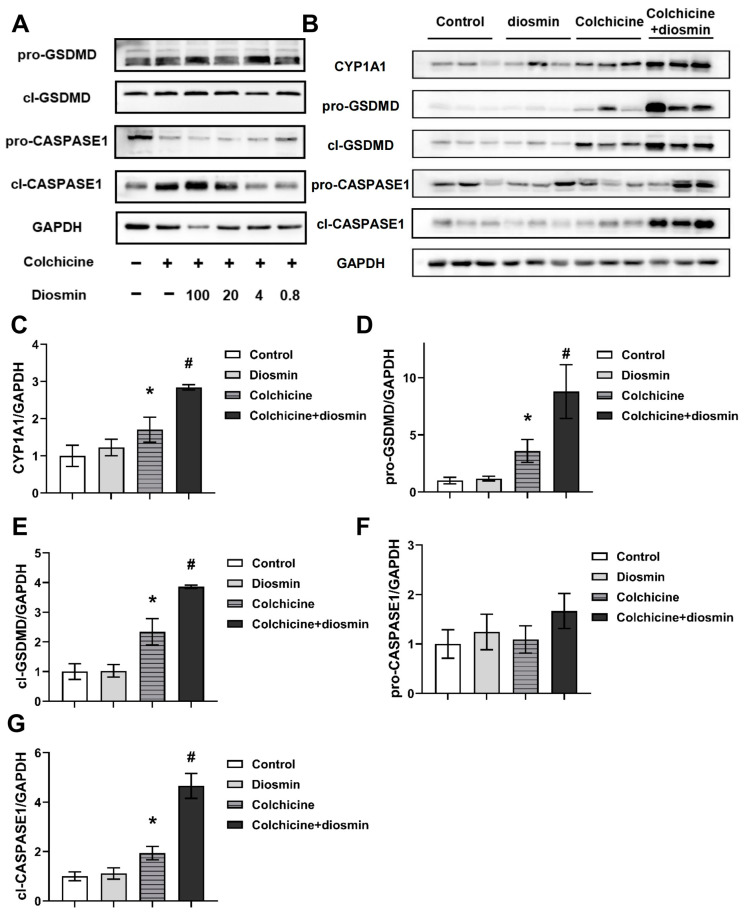
Regulation of CASPASE-1-mediated pyroptosis pathway by activating CYP1A1. Colchicine activated the expression of the CASPASE-1-GSDMD pathway, while diosmin up-regulated these proteins in a dose-dependent manner in primary hepatocytes (**A**) and in the liver (**B**). Relative intensities of immunoblots of CYP1A1 (**C**), pro-GSDMD (**D**), cl-GSDMD (**E**), pro-CASPASE1 (**F**) and cl-CASPASE1 (**G**). In the liver, total protein was normalized, and the results are shown as a bar graph after colchicine and diosmin management. All data are expressed as mean ± SD (*n* = 5); * *p* < 0.05 and # *p* < 0.05 were considered statistically significant when compared with the control group and colchicine group, respectively.

In the present study, we found that CASPASE-1-mediated pyroptosis was inhibited by inhibiting CYP1A1, and the CYP1A1 inducer diosmin was used to confirm this. In mice co-treated with colchicine and diosmin, the activation of the CASPASE-1-GSDMD pathway was aggravated (Figure 7). In response to diosmin in a dose-gradient manner, the CASPASE-1-GSDMD pathway was significantly up-regulated compared to the colchicine-only group in primary hepatocytes (Figure 7A). It has also been confirmed that diosmin enhanced colchicine-induced CASPASE-1-GSDMD pathway activation in animal experiments, which was demonstrated by the elevated protein levels of cl-CASPASE and cl-GSDMD (Figure 7B–G). Collectively, these results suggested that the CASPASE-1-GSDMD pathway played a role in hepatotoxicity induced by colchicine in C57BL/6J mice, possibly through the CYP1A1 enzyme.

## 3. Discussion

Previous studies reported that colchicine has excellent pharmacological activities, such as anti-inflammatory and anti-gout effects, but it can also induce severe side effects, such as gastrointestinal injury and liver damage at high doses in clinical treatment [19,20]. For the first time, we demonstrated that the inhibition of CYP1A1 alleviated liver injury induced by colchicine through the CASPASE-1-GSDMD-mediated pyroptosis pathway.

Colchicine is a microtubule inhibitor that can bind to tubulin dimers to prevent tubulin conversion and was found to prevent cell division during mitosis in vitro [21], which is one of the major factors causing liver toxicity. Previous studies on hepatotoxicity demonstrated that colchicine regulated the ubiquitin–proteasome pathway 3 to induce Mallory bodies [22]. In human hepatocytes, colchicine-induced microtubule destruction could disturb the stability of cytokeratin 18 [23]. Previous studies on oxidative stress and lipid peroxidation induced by colchicine focused on the brain [24]. In this study, the damaged liver induced by colchicine also showed oxidative stress and pyroptosis. However, it has also been reported that a low dose of colchicine inhibited oxidative stress in acute lung injury or thrombosis models [25,26], which may be due to the fact that the doses used for the colchicine model in this study were higher than those that exerted pharmacological effects. Abnormal oxidative balance in a colchicine model could cause lipid peroxidation and is related to liver damage. Furthermore, the activation of the CASPASE-1-GSDMD pathway induced pyroptosis both in vivo and in vitro in the colchicine model, which may play an important role in liver damage induced by colchicine. However, a low dose of colchicine inhibited the CASPASE-1 pathway in acute coronary syndrome patients [27], indicating that colchicine can regulate CASPASE-1 and that its dose is an key factor.

The major metabolic process of colchicine is demethylation which is regulated by CYP3A4 in vivo [13,28], while the role of CYP1A1 in colchicine metabolism has not yet been reported. A highlight of our study was that we found CYP1A1 also participated in the demethylation metabolism of colchicine. It has been reported that demethylation metabolism is a way of reducing colchicine toxicity in vivo [13,28], which is associated with promoting excretion, but whether CYP1A1 plays a role in colchicine-induced hepatotoxicity remains unknown. It was found that the inhibition of CYP1A1 could effectively improve liver injury, oxidative stress and pyroptosis induced by colchicine, while the activation of CYP1A1 aggravated colchicine-induced liver injury. This was inconsistent with the results of metabolic detoxification. Thus, we supposed that CYP1A1 might regulate colchicine-induced hepatotoxicity through other pathways.

CYP1A1 is a downstream target gene of aromatic hydrocarbon receptors (AHRs), involved in many drugs’ metabolism and associated with multiple toxicities [8,29]. CYP1A1 is a monooxygenase whose activation can lead to the overproduction of reactive oxygen species (ROS) and cause oxidative stress, which is a critical factor in hepatotoxicity [10]. Several reports have shown that AHRs are associated with pyroptosis and that the pyroptosis pathway is inhibited when AHRs or CYP1A1 is inhibited [30,31]. CYP1A1 is the key metabolic enzyme and target in many liver diseases, such as drug-induced liver injury and non-alcoholic fatty liver disease [9,32]. In fact, CYP1A1 is likely to be a downstream effector of colchicine. At a pharmacological dosage, colchicine reduces the level of TCDD-induced CYP1A1 [33]. As an anti-inflammatory drug, colchicine inhibited the activation of NLRP3 inflammasome as well as pyroptosis [34,35]. In this study, there were conventional hydrogen bonds and van der Waals, pi–cation and alkyl binding between CYP1A1 and colchicine (Figure 4), which suggested that colchicine may directly bind to CYP1A1 enzymes. This result supports our hypothesis that the hepatotoxicity of colchicine may be caused by the direct action of the CYP1A1 enzyme. However, the direct binding of colchicine to the CYP1A1 enzyme remains to be studied in the future.

Colchicine is a well-known substrate for CYP3A4 and P-gp. In clinical reports of colchicine, its interaction with CYP3A4/P-gp inhibitors has been reported [36,37]. Although the drug interaction between CYP1A1 and colchicine has not been reported so far, this study confirmed that colchicine affected the CYP1A1 enzyme. So, the potential toxicity of drug interaction between colchicine and CYP1A1 should be considered for clinical use in the future.

Interestingly, our present study found that colchicine may directly bind to CYP1A1, and a high dose of colchicine significantly up-regulated CYP1A1 and activated pyroptosis via the CASPASE-1 pathway in vivo and in vitro, which may be related to the disruption of oxidative balance. These findings collectively suggest that the hepatotoxicity of colchicine may be attributed to the CYP1A1-regulated signaling pathway. Therefore, our study points towards the possibility that the regulation of CYP1A1 may exert its beneficial effects against the liver damage induced by colchicine through the regulation of the CASPASE-1-GSDMD pathway, but further experiments are needed to confirm this.

## 4. Conclusions

**Figure 8:**
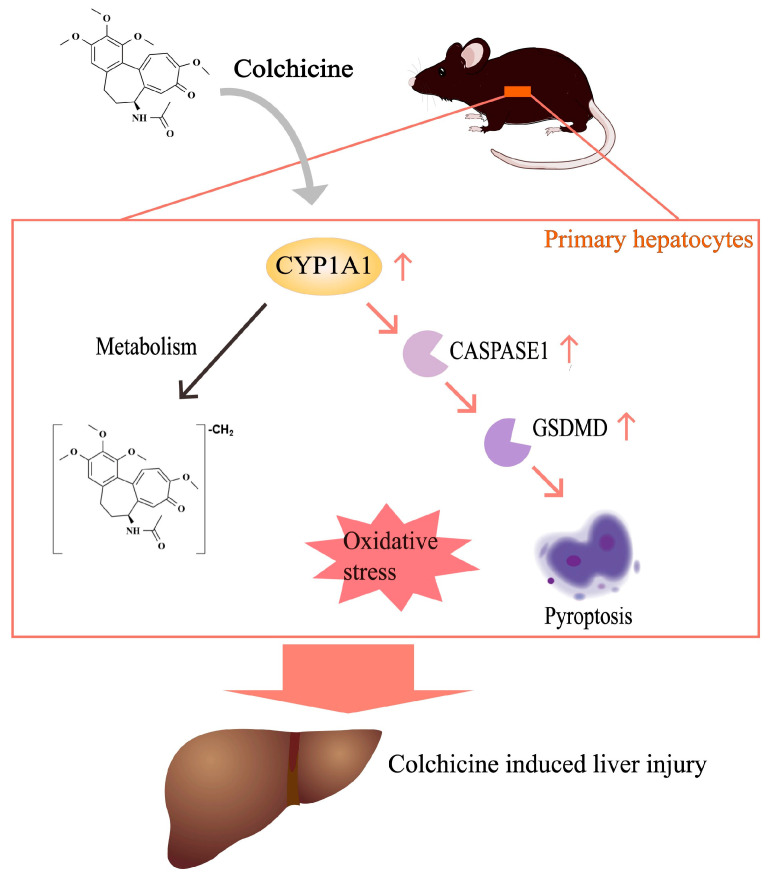
Proposed mechanism underlying the effect of CYP1A1 on colchicine-induced liver toxicity. CYP1A1 is involved in the demethylation metabolism of colchicine and regulates liver injury induced by colchicine, with a focus on oxidative stress and cell pyroptosis through the regulation of the CASPASE-1-GSDMD pathway.

In conclusion, CYP1A1 participated in the demethylation metabolism of colchicine and thus regulated colchicine-induced liver injury by alleviating oxidative stress and hepatocyte pyroptosis by mediating the CASPASE-1-GSDMD pathway (Figure 8). For the first time, CYP1A1 was shown to be involved in colchicine-induced hepatotoxicity, which may provide a potential target and strategy for the treatment of liver injury caused by colchicine.

## 5. Materials and Methods

### 5.1. Chemicals and Reagents

Colchicine (McLean, Shanghai, China), α-naphthoflavone and diosmin (Yuanye, Shanghai, China), and nicotinamide adenine dinucleotide phosphate (NADPH) (Sigma-Aldrich, St. Louis, MO, USA) were used in the study. Recombinant human CYPs were provided by the Research Institute for Liver Diseases Co., Ltd. (Shanghai, China).

### 5.2. Animals

The animal studies were approved by the Animal Care and Use Committee of West China Hospital, Sichuan University (20230412004, the ethics approval date was 12 April 2023). Chengdu Yaokang Biotechnology Co., Ltd. (Chengdu, China), provided the male C57BL/6J mice (18–22 g, 6 to 8 weeks old, No. SCXK (Sichuan) 2020-034). All animals were acclimated to the experimental environment for 1 week, and finally, the animals were anesthetized with CO_2_ and killed [38].

### 5.3. Animal Treatment

To study colchicine-induced liver injury, fifteen mice were divided into groups as follows: a colchicine group and a control group. It has been reported that a high dose of colchicine (4 mg/kg) can cause liver damage in mice [39]. Colchicine is used for gout at a dose of 0.025 mg/kg/d clinically, and not all patients who take colchicine have liver damage. A case has been reported in which liver damage occurred when colchicine was administered in large doses (>0.5 mg/kg) [40]. In order to establish a colchicine-induced liver injury model, 3 mg/kg (bio-equivalent to 7.5 times the clinical dose) and 5 mg/kg (bio-equivalent to 12.5 times the clinical dose) doses of colchicine were used to induce liver injury based on body surface area. The colchicine group was given the different doses of colchicine (3, 5 mg/kg), and the control group was given water by gavage for 3 consecutive days.

To demonstrate the role of CYP1A1 in colchicine-induced hepatotoxicity, α-naphthoflavone, the CYP1A1 inhibitor, was used as an intervention. Fifteen mice were divided into 3 groups (*n* = 5): a colchicine group, a colchicine + α-naphthoflavone group and a control group. Mice in the colchicine + α-naphthoflavone group were intraperitoneally injected with α-naphthoflavone (30 mg/kg, dissolved in corn oil) 2 h prior to colchicine (5 mg/kg) management for 3 days.

To investigate the role of CYP1A1 in colchicine-induced liver injury in mice, a CYP1A1 agonist (diosmin) was used. Twenty mice were divided into 4 groups (*n* = 5): a colchicine group, a colchicine + diosmin group, a diosmin group and a control group. Mice in the colchicine + diosmin group were given diosmin (100 mg/kg, dissolved in 0.5% CMC-Na) by gavage 2 h prior to colchicine administration (5 mg/kg). All mice in the control group received 0.5% CMC-Na prior to the administration of water, and the mice were sacrificed for sample collection 24 h after the final administration.

### 5.4. In Vitro Metabolism of Colchicine

Colchicine metabolism was determined using a previously described method [41], briefly described as follows: NADPH (1 mM), colchicine (25 µM), PBS (pH = 7.4) and each recombinant human CYP (2 pmol/mL) were included in a 200 µL incubation system. The control group was a system without NADPH or colchicine. The role of CYP1A1 in the metabolism of colchicine was investigated by co-culturing colchicine (25 µM) and α-naphthoflavone (10 µM) (the CYP1A1 inhibitor). Pre-cooled acetonitrile was used to terminate the reaction, and then centrifugation (4 °C, 12,000 rpm, 20 min) was performed to obtain the sample.

### 5.5. UHPLC-Q Exactive Plus MS Analysis

All samples were analyzed as shown in [17] and are briefly described in Supporting Information.

### 5.6. QPCR, WB, Histological, Immunohistochemistry and Biochemical Assessment

ALP, ALT and AST were measured in plasma, and MDA (Nanjing Jiancheng Bioengineering Institute, Nanjing, China) was measured in the liver. Histological characteristics were observed by preparing paraffin sections of liver tissue and performing hematoxylin and eosin (H&E) staining. Quantitative real-time PCR analysis, immunohistochemistry and Western blot analysis are described in Supporting Information [42,43,44].

### 5.7. Preparation of Primary Hepatocytes and Cell Viability Assay

The method of isolating primary mouse hepatocytes from mice (C57BL/6J) was that shown in the previous report [42], and it is briefly described in Supporting Information.

Cells were treated with a concentration gradient of colchicine (4–400 µM) and cultured for 48 h before being collected for quantitative real-time PCR analysis in the test. They were pre-treated with a concentration gradient of diosmin for 2 h, followed by the addition of colchicine (100 µM). The cells were harvested and used for protein extraction after incubation for 48 h.

### 5.8. Molecular Docking

Molecular docking was performed by using Discovery Studio 2020 software and the method described in [45]. Briefly, the 3D structure of the CYP1A1 enzyme (PDB ID: 4i8V) was obtained from the RCSB-PDB database. The protein was processed, the active site was identified and CDOCKER docking was performed after the molecular processing of α-naphthoflavone or colchicine.

### 5.9. Data and Statistical Analysis

LC-MS data were collected and processed using Thermo Xcalibur Qual Browser 4.3, and then MS/MS spectra were used to identify the chemical structures.

All data and images were analyzed unbiasedly. A random number was assigned to each group to ensure the investigator was blind to the pharmacological treatment grouping information. Statistical analysis was performed by using Graphpad Prism software 9.0, and *T*-test and one-way ANOVA were used for two- or multiple-group comparisons. *p* < 0.05 was considered statistically significant.

## Figures and Tables

**Table 1:** Results of colchicine metabolism in CYP recombinase enzyme.

	M1	M2	M3	M4	M5	M6	M7	M8
Control	0.00 ± 0.00	0.00 ± 0.00	0.00 ± 0.00	0.00 ± 0.00	0.00 ± 0.00	0.00 ± 0.00	0.00 ± 0.00	0.00 ± 0.00
CYP1A1	3.91 ± 3.16	4.51 ± 2.24	4.92 ± 1.48	6.99 ± 0.30	6.87 ± 0.00	8.78 ± 1.10	8.53 ± 0.61	9.32 ± 5.12
CYP1A2	6.36 ± 0.19	0.00 ± 0.00	7.83 ± 0.27	6.72 ± 0.44	8.33 ± 0.00	8.69 ± 0.17	5.42 ± 0.50	8.67 ± 3.80
CYP1B1	4.56 ± 1.73	2.53 ± 0.65	8.08 ± 0.40	7.00 ± 0.10	7.27 ± 2.20	7.14 ± 0.77	4.80 ± 0.73	7.48 ± 2.17
CYP2A6	5.39 ± 0.90	2.64 ± 0.16	8.53 ± 1.02	6.66 ± 2.12	7.26 ± 8.64	8.25 ± 0.69	4.07 ± 0.65	4.23 ± 0.62
CYP2B6	6.90 ± 0.52	4.42 ± 0.21	8.37 ± 0.52	7.77 ± 0.57	7.86 ± 0.00	9.86 ± 0.58	12.35 ± 0.43	10.13 ± 3.38
CYP2C19	8.35 ± 0.65	6.27 ± 0.30	7.68 ± 0.04	6.69 ± 1.91	8.63 ± 1.05	6.74 ± 0.36	4.10 ± 0.63	7.66 ± 2.52
CYP2C8	6.82 ± 0.03	3.69 ± 0.02	7.94 ± 0.13	7.21 ± 0.40	7.37 ± 0.00	6.90 ± 1.01	10.77 ± 0.42	8.61 ± 4.55
CYP2C9	13.68 ± 5.29	5.57 ± 1.45	6.83 ± 0.42	6.44 ± 0.02	7.59 ± 0.00	7.33 ± 0.35	9.27 ± 0.35	6.12 ± 3.22
CYP2D6	7.91 ± 0.60	3.23 ± 0.56	7.20 ± 0.32	11.27 ± 3.15	7.65 ± 11.17	7.59 ± 0.36	0.00 ± 0.15	0.00 ± 0.00
CYP2E1	4.77 ± 2.02	3.69 ± 0.51	8.11 ± 0.05	6.93 ± 0.40	7.32 ± 0.00	8.09 ± 0.37	7.85 ± 0.54	6.78 ± 0.72
CYP3A4	14.93 ± 1.41	47.73 ± 5.45	7.46 ± 0.07	12.18 ± 0.70	7.72 ± 15.62	6.55 ± 0.65	14.09 ± 0.08	12.27 ± 1.54
CYP3A5	9.93 ± 0.49	12.14 ± 0.15	8.56 ± 0.20	7.36 ± 0.44	8.12 ± 0.00	8.06 ± 0.09	14.87 ± 0.31	11.08 ± 1.09
CYP4A11	6.48 ± 0.33	3.57 ± 0.39	8.49 ± 0.07	6.78 ± 0.33	8.01 ± 0.00	6.01 ± 0.22	3.87 ± 0.04	7.65 ± 1.95

Notes: The role of individual CYPs in colchicine metabolism was determined by using cDNA-expressed CYPs (control, CYP1A1, 1A2, 1B1, 2A6, 2B6, 2C19, 2C8, 2C9, 2D6, 2E1, 3A4, 3A5 and 4A11). “M” represents the metabolite. All samples were analyzed by UHPLC-Q Exactive plus MS. The sum of the total peak areas of each metabolite of colchicine from all the CYPs was set as 100%, and the data in the table are the peak areas of each CYP enzyme after incubation as a percentage of the total peak areas. All data are expressed as mean ± SD (*n* = 2).

## Data Availability

The data presented in this study are available in this article.

## Supplementary Materials

The following supporting information can be downloaded at: https://www.mdpi.com/article/10.3390/toxins16010035/s1, Figure S1: Colchicine caused hepatotoxicity in a dose-dependent manner. (A) Gross anatomy of the liver after colchicine treatment. (B) Liver inflammatory injury as observed by H&E staining was shown by black arrow arrowheads and hyalinization as observed by H&E staining was shown by the black circle after a high dose of colchicine management. Alanine aminotransferase (ALT) (C), aspartate aminotransferase (AST) (D) and alkaline phosphatase (ALP) (E) enzyme levels were significantly increased after colchicine management, respectively. (F) Malondialdehyde (MDA) levels after colchicine administration. (G) Gene expression level of CASPASE-1 in liver after colchicine treatment. All data were expressed as mean ± SD (*n* = 5). * *p* < 0.05, compared with the control group, there was significant difference as indicated; Figure S2: Metabolic map of colchicine; Table S1: Primer sequences for QPCR.

## Abbreviations

AHR, aromatic hydrocarbon receptor; ALP, alkaline phosphatase; ALT, alanine transaminase; AST, aspartate transaminase; CYP, cytochrome P450; H&E, hematoxylin and eosin; MDA, malondialdehyde; ROS, reactive oxygen species.
