# Supplementary material for: Inhibition of CYP1A1 Alleviates Colchicine-Induced Hepatotoxicity
Source: Toxins (Basel). 2024 Jan 9;16(1):35. doi: 10.3390/toxins16010035 (PMC10818746; doi:10.3390/toxins16010035)
Supplement: Supplementary file 1 [file toxins-16-00035-s001.zip › toxins-2743266-supplementary.pdf]

Supplementary Materials: Inhibition of CYP1A1 Alleviates Colchicine-Induced Hepatotoxicity

Ruoyue Huang, Jingyi Duan, Wen Huang, Yan Cheng, Beiwei Zhu and Fei Li

Supplementary materials and methods

Reference numbers refer to the main text list

UHPLC-Q exactive plus MS Analysis

All samples were analyzed on a liquid chromatography system including a Vanquish autosampler, Vanquish detector, Vanquish pump (Thremo Fisher Scientific, Santa Clara, CA). Samples were separated on an ACQUITY UPLC HSS T3 column (2.1×100 mm, 1.8 mm, Waters, USA). The injection volume: 5 μL, and the flow rate was 0.3 mL/min. Phase A was H_2O containing 0.01% formic acid, and phase B was acetonitrile containing 0.01% formic acid. The elution gradient was as follows: 0-12 min, 2-98% B; 12-14 min, 2% A; 14-16 min, 98% A. Other parameters are as follows: column temperature 45 ℃, flow rate of collision gas and dry gas 9 L/min. Capillary voltage 3.5 kV, temperature 350 ℃, atomizer pressure 35 psi. Vanquish UHPLC-Q Exactive plus MS (Thermo Fisher, USA) was collected in both positive modes, operating in full scan mode with 60-900 m/z.

Preparation of primary hepatocytes

Primary mouse hepatocytes were isolated from male C57BL/6J mice as shown in previous literature [42]. The liver was perfused through the portal vein with Hanks solution twice (the first solution contains 1% penicillin/streptomycin, the second solution contains 0.05% collagenase IV) after mice were anesthetized with ether, and then they were killed by exsanguination. Hepatocytes were harvested by centrifugation three times (The first centrifugal condition is 500 rpm for 3 minutes, the second and third centrifugal condition is 1500 rpm for 5 min) after the perfused liver was passed through 100 filter sizes, and they were re-suspended in Dulbeco Modified Eagle Medium (DMEM) (Hyclone, USA). Cells were collected and seeded at a density of 2×10^5 cells ml^{-1} in plates and cultured overnight in William E (Gibco, USA) medium (0.5 U/mL insulin, 1% penicillin/streptomycin, 10% FBS and 0.4 μg/mL dexamethasone) at 37 ℃ in a humidified Air /CO2 Incubator (5% V/V).

Quantitative real-time PCR analysis

QPCR were performed as described previously [43]. Briefly, Trizol reagent (Life Technologies, Carlsbad, CA, USA) and SYBR Green PCR master mixture (Vazyme, Nanjing, China) were used for the experiment. The qPCR was performed in the CFX Connect real-time system (Bio-Rad Laboratories, Hercules, CA, USA), with 18S was used as internal reference, and the qPCR primer sequence was provided in Table S1.

Western blot analysis

The methods for Western blot analysis were described in previous literature [44]. Briefly, RIPA (POO13B, Beyotime Biotech), BCA kit (P0009, Beyotime Biotech), and 5% BSA (A8020, Solarbio) buffer were used for the experiment. The following antibodies were used: CASPASE-1 (Santa Cruz Biotechnology, Cat# sc-56036), CYP1A1 (Santa Cruz Biotechnology, Cat# sc-25304), GAPDH (Cell Signaling Technology, Cat# 2118), GSDMD (Santa Cruz Biotechnology, Cat# sc-393656), Goat anti-Mouse IgG-HRP Antibody (HUABIO, Cat# HA1006) and Goat anti-Rabbit IgG Goat Polyclonal Antibody (HUABIO, Cat# HA1001).

Immunohistochemistry

Immunohistochemistry was determined by used the previously described method [43]. Briefly, liver tissue sections underwent antigen repair, and incubated with CYP1A1 antibody overnight at 4 ℃. The secondary antibody was added for incubation after washing with PBS, and 3,3-N-Diaminobenzidine Tertrahydrochloride (DAB) was used for color development in the end.

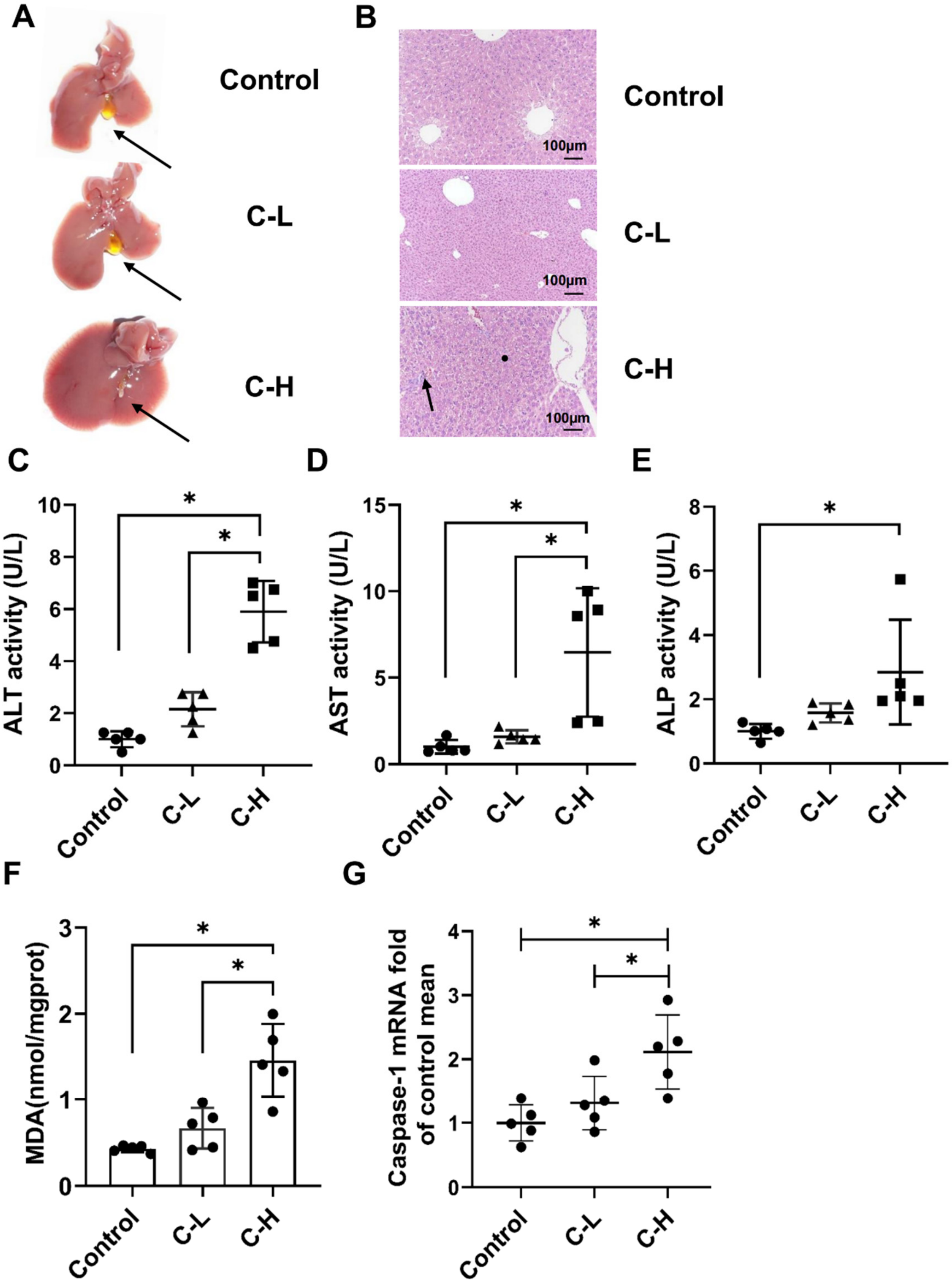

Figure S1. Colchicine caused hepatotoxicity in a dose-dependent manner. (A) Gross anatomy of the liver after colchicine treatment. (B) Liver inflammatory injury as observed by H&E staining was shown by black arrow arrowheads and hyalinization as observed by H&E staining was shown by the black circle after a high dose of colchicine management. Alanine aminotransferase (ALT) (C), aspartate aminotransferase (AST) (D) and alkaline phosphatase (ALP) (E) enzyme levels were significantly increased after colchicine management, respectively. (F) Malondialdehyde (MDA) levels after colchicine administration. (G) Gene expression level of Caspase-1 in liver after colchicine treatment. All data were expressed as mean ± SD (n = 5). * $p < 0.05$, compared with the control group, there was significant difference as indicated.

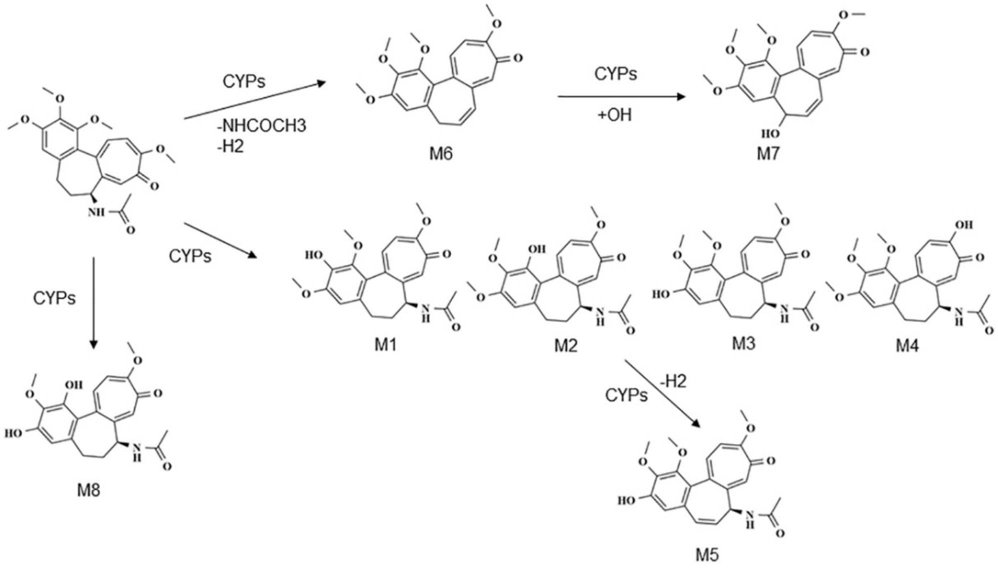

Figure S2. Metabolic map of colchicine.

Table S1. Primer sequences for QPCR.

Gene	Abbreviation	Sequence
Cytochrome P450 family 1 subfamily A member 1	*Cyp1a1*	CAATGAGTTTGGGGAGGTTACTG
		CCCTTCTCAAATGTCCTGTAGTG
Caspase-1	*Caspase-1*	TACCTGGCAGGAATTCTGGA
		AGTCCTGGAAATGTGCCATC
18s	*18s*	ATTGGAGCTGGAATTACCGC
		CGGCTACCACATCCAAGGAA
